# Bacterial-Based Strategies to Hydrolyze Gluten Peptides and Protect Intestinal Mucosa

**DOI:** 10.3389/fimmu.2020.567801

**Published:** 2020-11-03

**Authors:** Fernanda Cristofori, Ruggiero Francavilla, Daniela Capobianco, Vanessa Nadia Dargenio, Simone Filardo, Paola Mastromarino

**Affiliations:** ^1^ Interdisciplinary Department of Medicine—Pediatric Section, Università di Bari Aldo Moro, Bari, Italy; ^2^ Department of Public Health and Infectious Disease, Università La Sapienza di Roma, Rome, Italy

**Keywords:** celiac disease, microbiota, probiotics, gluten hydrolysis, gluten digestion

## Abstract

Gluten is a mixture of proteins highly resistant to hydrolysis, resulting in the emergence of toxic peptides responsible for gluten-related disorders. Currently, a gluten-free diet (GFD) is the unique proven therapy for celiac disease (CD). Several research groups and pharmaceutical companies are developing new nondietetic therapeutic strategies for CD. Probiotics are viable microorganisms thought to have a healthy effect on the host. The proteolytic mechanism of lactic acid bacteria comprises an extracellular serine protease, di- and oligopeptide-specific transport systems, and several intracellular peptidases that might affect gluten degradation. Therefore, probiotic supplementation is an attractive therapy because of its possible anti-inflammatory and immunomodulatory properties. Several studies have been performed to assess the effectiveness of various specific probiotic strains, showing positive effects on immune-modulation (inhibition of pro-inflammatory cytokine TNF-α) restoring gut microbiota and decrease of immunogenic peptides. The present review aims to summarize the current knowledge on the ability of probiotic strain (single or mixtures) to digest gliadin peptides in vitro and to modulate the inflammatory response in the gut.

## Introduction

Celiac disease (CD) is an autoimmune enteropathy that occurs in genetically predisposed individuals who develop an immune response after gluten ingestion (1). The prevalence of this condition, estimated at around 1%, is increasing worldwide. The gold standard in CD treatment relies on a gluten-free diet (GFD) that needs to be strict and lifelong and should be directed by a specialist nutritionist (2); although novel nondietetic treatments are under study, GFD is the only available therapy for CD at present.

CD is a polygenic disease with a strict gene environmental interaction, and recently, the role of gut microbiota (GM) has become of primary interest. Available literature supports the theory that modification in GM is related to many chronic inflammatory diseases, including obesity, diabetes, inflammatory bowel disease, and CD (3). The role of the GM in CD is supported by evidence that germ-free mice develop a gluten-related pathology whose severity depends on the type of microbial colonization in their guts (4), hence the idea that GM modulation with the use of probiotics can be used for therapeutic purposes in the course of CD.

The information describing the gut microbiota in CD patients has been derived from both fecal samples and duodenal mucosa biopsies generally in children with CD. Various authors have found contrasts in the microbial composition of biological samples when comparing active CD to patients adherent to the GFD or healthy controls. The following findings (demonstrated by more than two independent evidences) supports the association between CD and GM: 1) increased Bacteroidetes in biopsies and stools of active and inactive CD, 2) decreased fecal *Bifidobacterium* spp. in active and inactive CD, 3) increased Proteobacteria in biopsies and stools of active CD, and 4) increased *Staphylococcus* in biopsies and stools of active CD (5).

“Probiotics are ‘live microorganisms’ that confer, if administered in adequate amounts, benefit(s) to the host health” (6). Probiotic mechanisms of action include modulation of GM, suppression of potentially harmful microorganisms (producing antimicrobial or other factors, thus suppressing growth or competing for mucosal receptors of pathogens), regulation of the immune system, and mucosal barrier strengthening.

Therefore, several studies look at the possible use of probiotics in CD as complementary treatment to modulate the microbiome or detoxify gluten (7).

## Probiotics and Wheat Deglutination

Gliadin and glutenin (gluten immunogenic peptides) are the major antigens leading to intestinal damage in CD. Gliadin and glutenin are rich in proline and glutamine residues and are able to resist gastric, pancreatic, and intestinal proteolysis. Some of these immunogenic peptides can induce the inflammatory process associated with CD (8, 9).

The human GM includes bacterial species able to degrade gluten peptides, affecting their toxicity (10–12). However, the microbiota may either be protective against or contribute to the development of CD through the generation of harmful immunogenic peptides (13, 14).

Indeed, strains of *Bacteroides fragilis*, isolated from the GM of CD patients, display gliadin-hydrolyzing activity, some of them giving rise to immunogenic peptides and, hence, inducing inflammatory cytokine production by intestinal epithelial cells (13). Moreover, *Pseudomonas aeruginosa* isolates, recovered from the duodenum of CD patients, produced, via their elastase activity, several peptides that elicited the activation of gluten-specific T-cells in these patients. By contrast, *Lactobacillus* spp, isolated from healthy individuals, degraded *P. aeruginosa*-modified peptides and reduced their immunogenicity (14).

Lactobacilli and bifidobacteria are considered essential intestinal microbial genera that have beneficial effects on human health and are widely used in the formulation of probiotic products. Therapy with probiotics, containing bacteria capable of degrading gluten, is a possible new strategy for the complementary treatment of CD patients. Indeed, a compelling approach could consist of the in situ detoxification of gluten by resident intestinal bacteria through their metabolic activity.

**Figure 1:**
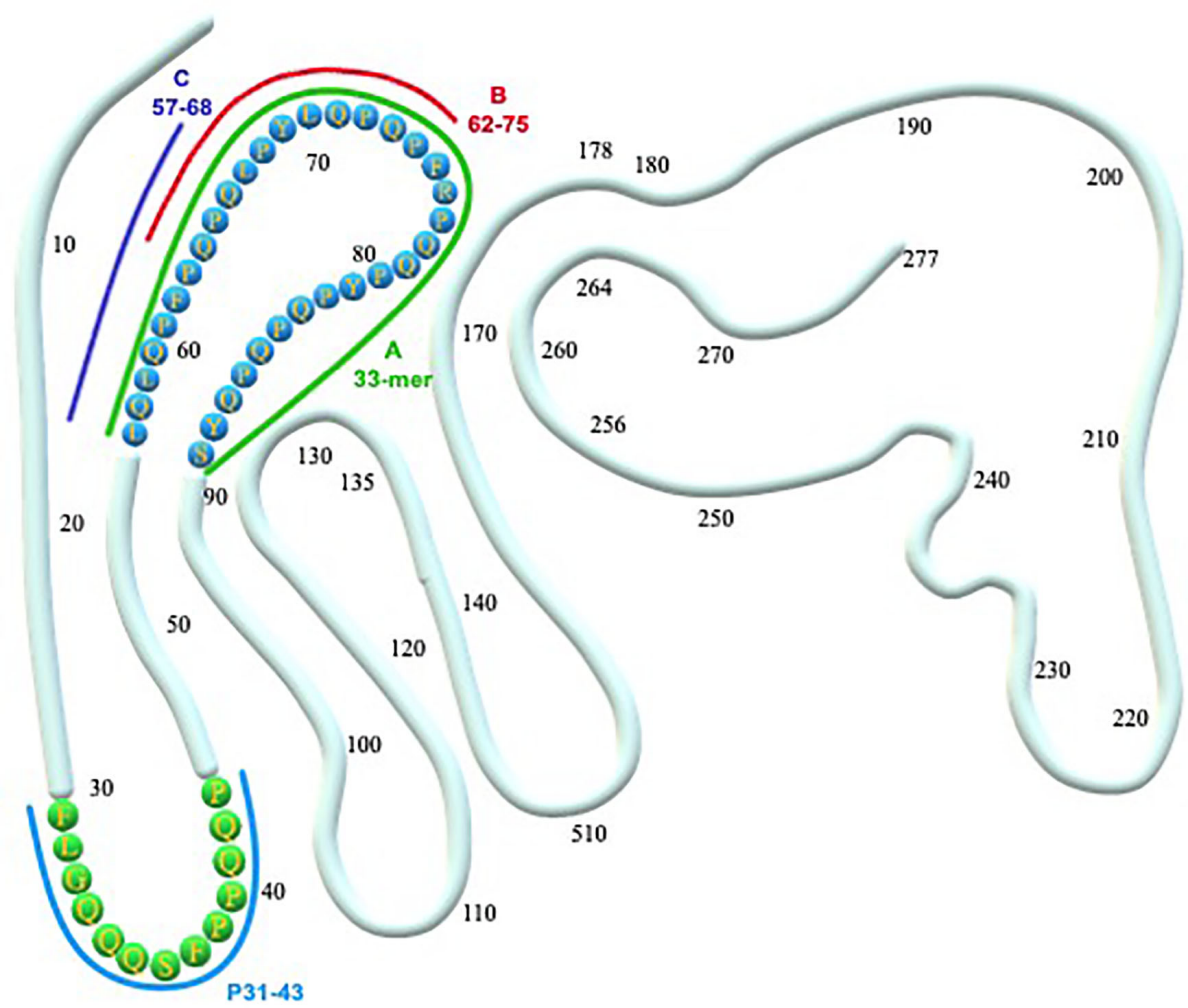
α-gliadin structure and toxic epitopes (33-mer and p31-43). A, B, C represent the peptides hydrolyzed by a different mixture of probiotic strains. **(A)** Hydrolyzed by a mixture of L. paracasei, L. plantarum, Bifidobacterium animalis subsp. lactis, B. breve Bbr8, B. breve BL10 (18). **(A**, **B)** Hydrolyzed by a mixture of L. alimentarius, L. brevis, L. sanfranciscensis, L. hilgardii (15); mixture of Streptococcus thermophilus, L. plantarum, L. acidophilus, L. casei, L. delbrueckii subsp. bulgaricus, Bifidobacterium breve, B. infantis, B. longum (16). **(A–C)** Hydrolyzed by a mixture of L. casei, L. delbrueckii subsp. bulgaricus, L. paracasei LPC01 and BGP2, L. plantarum BGP12, LP27, LP35, LP40, LP47, and SP1 (17).

Several publications have reported the results of in vitro and in vivo studies on lactobacilli and bifidobacteria strains capable of hydrolyzing gluten proteins (**Table 1**, **Figure 1**) reducing their toxicity and/or inflammatory effect. Most of the studies are carried out on different strains and species of lactobacilli, identified as the principal gluten-metabolizing bacteria in the gut (10, 11). Indeed, it is suggested that lactobacilli possessed a competitive advantage on other microorganisms due to the property to produce nitrogen from gluten (10): Extracellular and cell wall–associated proteases cleave proteins into oligopeptides, carried across the cell membrane and then hydrolyzed by intracellular peptidases (21).

**Table 1:** *In vitro* and *in vivo* studies on lactobacilli and bifidobacteria strains capable of hydrolyzing gluten proteins reducing their toxicity and/or inflammatory effect.

Microorganism	Identified enzymes	Hydrolysis effect	Biological effect	Reference
Mixture of *Lactobacillus alimentarius*, *L. brevis*, *L. sanfranciscensis*, *L. hilgardii*	Iminopeptidase, dipeptidyl-peptidase, prolylendopeptidase, prolidase, prolinase, aminopeptidase P	Complete hydrolysis of α2-gliadin-derived epitopes 62–75 and 33-mer	No alteration of intestinal permeability after ingestion of bread produced with wheat flour fermented with lactobacilli in CD patients on a GDF	Di Cagno et al. (15)
Mixture of *Streptococcus thermophilus*, *Lactobacillus plantarum*, *L. acidophilus*, *L. casei*, *L. delbrueckii* subsp. *bulgaricus*, *Bifidobacterium breve*, *B. infantis*, *B. longum*	Proline iminopeptidase, aminopeptidase N and A, dipeptidase, prolinase, prolidase, dipeptidyl peptidase, tripeptidase, prolylendopeptidase endopeptidase	Complete hydrolysis of α2-gliadin-derived epitopes 62–75 and 33-mer	Reduced alteration of gliadin-induced increase of intestinal cell cultures permeability	De Angelis et al. (16)
Mixture of *L. casei*, *L. delbrueckii* subsp. *bulgaricus*, *L. paracasei* LPC01 and BGP2, *L. plantarum* BGP12, LP27, LP35, LP40, LP47, and SP1	Aminopeptidase N, iminopeptidase, prolyl endopeptidyl peptidase, tripeptidase, prolidase, prolinase, dipeptidase	Complete hydrolysis of α9-gliadin peptide 57-68, 33-mer, A-gliadin peptide 62-75, γ-gliadin peptide 62-75 Absence of immunogenic peptides after hydrolysis of the wheat bread gluten	Reduction to basal level of IL-2, IL-10, and IFN-γ increase induced by untreated wheat bread gluten in duodenal biopsy specimens from CD patients	Francavilla et al. (17)
Mixture of *Lactobacillus paracasei*, *L. plantarum*, *Bifidobacterium animalis* subsp. *lactis*, *B. breve* Bbr8, *B. breve* BL10	–	Hydrolysis of gliadin peptides generated by digestive proteases into smaller fragments. Hydrolysis of 33-mer	Inhibition of the inflammatory state and disruption of tight junctions induced by gliadin in intestinal epithelial cells.	Giorgi et al. (18)
*Bifidobacterium animalis*, *B. longum*, *B. bifidum*	–	Different peptide patterns with lower molecular mass compared to those noninoculated with bacteria	Reduction of the cytotoxic effect and inhibition of NF-kB activation and TNF-α production induced by gliadin in intestinal epithelial cells especially by *B. longum*.	Laparra et al. (19)
*Bifidobacterium bifidum*, *B. longum*, *B. breve*, *B. animalis* individual species and as mixture	–	Hydrolysis of gluten in different peptide patterns	Reduction of the cytotoxic effect and inhibition of NF-kB activation and TNF-α production induced by gluten in intestinal epithelial cells especially by *B. longum* and the *Bifidobacterium* consortium	Castilho de Almeida et al. (20)

A mixture of *Lactobacillus alimentarius* 15M, *Lactobacillus brevis* 14G, *Lactobacillus sanfranciscensis* 7A, and *Lactobacillus hilgardii* 51B, chosen for their distinct hydrolysis profiles toward wheat gliadins, were capable of fully hydrolyzing the fragment 62-75 of A-gliadin and the 33-mer peptide (15). The probiotic mixture showed iminopeptidase, dipeptidyl-peptidase, prolyl endopeptidase, prolidase, prolinase, and aminopeptidase P enzymatic activities. The effectiveness of lactobacilli on wheat deglutination was verified by in vivo acute challenge in CD patients on a GDF. As a result, these patients did not display any gut permeability impairment after eating bread produced with lactobacilli-fermented wheat flour.

Subsequent studies have confirmed that the simultaneous presence of proteases with different specificities is necessary to obtain complete hydrolysis of the toxic peptides derived from gliadin. The combined activity of general aminopeptidase type N (PepN; EC 3.4.11.11), endopeptidase (PepO; EC 3.4.23), and prolyl endopeptidyl peptidase (PEP; EC 3.4.21.26), obtained from selected sourdough lactobacilli, induced the hydrolysis of the CD immunogenic 33-mer peptide into five small peptides (22). Overall, five peptidases were necessary to fully metabolize the 33-mer and other synthetic immunogenic peptides (22). Previously, the same group demonstrated that a probiotic preparation, containing *Streptococcus thermophilus*, *Lactobacillus plantarum*, *Lactobacillus acidophilus*, *Lactobacillus casei*, *Lactobacillus delbrueckii* subsp. *bulgaricus*, *Bifidobacterium breve*, *Bifidobacterium infantis*, and *Bifidobacterium longum*, reduced wheat flour toxicity during sourdough fermentation for an extended period (16). However, the ability of probiotic microorganisms to hydrolyze gliadin peptides was missing when the individual strains were assayed. These studies suggest that no individual bacterial strain have the whole spectrum of peptidases required to degrade the different peptides contributing to CD.

On the other hand, the healthy GM is a very complicated ecosystem characterized by several bacterial species that can be involved in the degradation of gliadin peptides through enzymes that are not produced by humans. Therefore, the different kind of proteases found in the gut, each with its specificity, could be responsible for the complete hydrolysis of toxic gliadin peptides.

Recently, several lactobacilli strains (*L. casei* BGP93; *L. delbrueckii* subsp. *bulgaricus* SP5; *L. paracasei* LPC01, BGP1, and BGP2; *L. plantarum* BG112, BGP12, LP27, LP33, LP35, LP36, LP39, LP40, LP42, LP47, and LP32; *L. rhamnosus* SP1; and *L. reuteri* DSM17938) have been evaluated for the ability to hydrolyze immunogenic gluten peptides. A highly variable peptidase activity has been observed in different strains (i.e., iminopeptidase, aminopeptidase N, prolidase, prolyl endopeptidyl peptidase, prolinase, tripeptidase, and dipeptidase). Ten strains, selected to provide the best global peptidase activity needed for completely degrading the immunogenic gluten peptides involved in CD, were pooled and tested for digestion of gliadin peptides. The probiotic mixture was shown to fully hydrolyze immunogenic epitopes, such as the gliadin 33-mer peptide, the peptide spanning residues 57 to 68 of the a9-gliadin (α-gliadin peptide 57-68), A-gliadin peptide 62-75, and γ-gliadin peptide 62-75. The selected lactobacilli mixture strongly hydrolyzed the wheat bread gluten (ca. 18,000 ppm) to less than 10 ppm following a treatment lasting 360 min under simulated GI conditions. The gluten-detoxifying activity was confirmed by culturing duodenal biopsy specimens, obtained from CD patients following a GFD with digested baker’s yeast wheat bread in either the absence or the presence of probiotic lactobacilli. The levels of IL-2, IL-10, and IFN-γ were overexpressed as a consequence of pepsin-trypsin (PT) wheat bread digestion products that were not treated with the probiotic strains. In contrast, the level of cytokines was similar to the baseline value in duodenal biopsy specimens exposed to PT digestion products from wheat bread, containing the selected probiotic strains (17).

Recently, our group studied the capacity of a probiotic mix (*Lactobacillus paracasei*, *Lactobacillus plantarum*, *Bifidobacterium animalis* subsp. *lactis*, *Bifidobacterium breve* Bbr8, *Bifidobacterium breve* BL10) to hydrolyze gluten peptides following the digestion of gliadin and to modify the pro-inflammatory state as well as the gliadin-induced epithelial modification in the gut (18). The tested lactobacilli and bifidobacteria strains were, indeed, capable of hydrolyzing the fragments of gliadin, produced by digestive proteases, into smaller fragments. In particular, the number of peptides with a molecular weight lower than 3 kDa was significantly higher in the PT-gliadin plus bacteria as compared to PT-gliadin. Moreover, the probiotic strains could degrade the 33-mer immunotoxic peptide in case the compound was the only amino-acid source. Digestion of PT fragments from gliadin with the probiotic strains counteracted the inflammatory state and the disruption of tight junctions induced by PT-gliadin in intestinal epithelial cells.

*In vitro* and *in vivo* studies demonstrate that some *Bifidobacterium* species possess the ability to hydrolyze the toxic gliadin-derived peptides as well as to inhibit the related inflammatory response in intestinal epithelial cells. Different *Bifidobacterium* strains (*B. animalis* IATA-A2, *B. longum* IATA-ES1, *B. bifidum* IATA-ES) added to a mixture of gliadins during simulated intestinal digestion, produced different peptide patterns with lower molecular mass compared to those noninoculated with bacteria (19). Gliadin-mediated NF-kB activation (responsible for the activation of the pro-inflammatory pathway) and TNF-α levels significantly decreased in intestinal epithelial cells cultures exposed to gliadin digestions inoculated with all bifidobacterial strains mostly in those exposed to digests added with *B. longum*.

The administration of the same *B. longum* strain has also been shown to decrease jejunal TNF-α levels in an *in vivo* model of gliadin-induced enteropathy (23). Recently, the effect of *Bifidobacterium* species, namely *B. bifidum*, *B. animalis*, *B. breve*, and *B. longum*, on the hydrolysis of unmetabolized gluten proteins as well as on the related toxic effect and immune responses elicited by the resulting peptides, has been investigated (20). The different individual species and a mixture of the four *Bifidobacterium* species resulted in diverse peptide patterns, deriving from the principal wheat protein classes, namely gliadins and glutenins, and presenting from 15 to 40 long amino-acid residues. *B. longum* showed the highest proteolytic activity toward gluten proteins. In addition, *B. longum* and the *Bifidobacterium* consortium possessed a proteolytic activity that acted mostly on gliadin hydrolysis, whereas *B. animalis* favored glutenin digestion. The resulting peptides from the *Bifidobacterium* cultures had a significantly decreased cytotoxic effect on intestinal cell cultures, especially those produced by *B. longum*, as compared to gluten-digested peptides from noninoculated samples. The immune response elicited by the gluten-digested peptides from the *Bifidobacterium* cultures was studied by assaying NF-kB activation and TNF-α and IL-1β expression in cell cultures. As a result, samples containing the peptides from the *Bifidobacterium* cells displayed a significantly reduced TNF-α expression and NF-kB activation as compared to the positive controls. Furthermore, the decrease in TNF-α levels and NF-kB activation depended on the *Bifidobacterium* species utilized. In this regard, *B. longum* and the *Bifidobacterium* consortium were the most effective in reducing TNF-α, and the *B. bifidum* strain was the least active.

A different approach can also be used to ameliorate gluten intolerance, such as the use of gluten-digesting bacteria to detoxify gluten before the administration to patients. As previously described, the major gluten protein gliadin can be hydrolyzed by lactic acid bacteria under fixed processing conditions (15, 16). The use of selected sourdough lactic acid bacteria cultures as starters for fermentation has been suggested as a way to eliminate the risk for gluten contamination. A sourdough containing *Lactobacillus plantarum* CF1 and *Lactobacillus sanfranciscensis* LS40 and LS41, chosen for their proteolytic activity, was used for the production of gluten-free bread (24). Gluten was added to the ingredients before fermentation to simulate potential contamination, and as a result, a substantial reduction of gluten concentration (from 400 ppm to below 20 ppm) was observed. In clinical trials, the administration of fermented foods to CD subjects was also proved safe (25, 26). Baked goods made of wheat flour, rendered gluten-free (gluten to <10 ppm) during sourdough fermentation through specific lactobacilli and fungal proteases, were nontoxic to young CD patients (25). Similarly, a 60-day diet of baked goods made from this hydrolyzed form of wheat flour showed no toxicity to CD patients (26).

Our group recently described a protocol for the manufacture of reduced-gluten bread and pasta via fungal proteases as well as selected sourdough lactic acid bacteria. The efficacy of these products (containing <50% gluten content) was tested in a randomized, double-blind, crossover-controlled trial of 24 patients with irritable bowel syndrome (IBS) (compared to traditional bread and pasta). We demonstrated that, while consuming bread and pasta with reduced gluten content, IBS patients had significant symptom improvement as measured by the visual analogue scale score (p = 0.042) (27).

At present, in vivo studies fail to provide evidence that probiotic bacteria are able to degrade gluten peptides at a rate capable of guaranteeing that no immunogenic gluten peptides survive in the gut to stimulate CD4 T cells.

## Probiotics in CD Patients

The theoretical possibility of toxic gliadin peptide digestion led to the hypothesis that probiotics might be useful in patients with CD. Most of the studies have been performed in patients on GFD, and only a few have been carried out in patients on a gluten-containing diet (GCD).

The effect of *Bifidobacterium infantis* Natren life start strain (NLS-SS) was evaluated in a randomized controlled trial (RCT) on 22 CD patients on GCD (12 g of gluten/day). The authors demonstrate that this specific strain alleviates GI symptoms (gastroesophageal reflux, constipation, and indigestion measured by gastrointestinal symptom rating scale) without any influence on CD serology, gut permeability, growth factors, and cytokines (28). The authors hypothesized that the favorable symptomatic observed effect might be related to an effect on the innate immunity. The mechanisms on innate immunity markers were tested assessing human α-defensin 5 (HD5) expression as well as Paneth cells and macrophage counts in duodenal biopsies in three different groups of patients. Thirty-six patients with active CD were blind randomized to *Bifidobacterium infantis* (12 patients; group 1) or placebo (24 patients; group 2); 5 CD patients composed the third group after 1 year of GFD. In this study, the authors demonstrated that *Bifidobacterium infantis* NLS-SS reduces Paneth cell and HD5 expression. On the contrary, GFD induces a more significant reduction of macrophage in duodenum than *B. infantis* (29).

Håkansson et al. tested the immunomodulatory effect of *L. plantarum* HEAL9 and *L. paracasei* 8700:2 in pediatric patients with CD autoimmunity before diagnosis and GFD. They studied 78 children with CD autoimmunity for 6 months (40 received the two lactobacilli and 38 placebo). The authors found significant changes in the peripheral immune response implicated in T-cell regulation only in children that received placebo. The results show that *L. paracasei* and *L. plantarum* may play a regulatory role on the peripheral immune response in CD. Moreover, the authors found a more significant reduction of the levels of IgA-tTG in the probiotic (*p* = 0.013) compared to placebo (30).

The effect of the administration of a mixture of two *Bifidobacterium breve* strains (B632 and BR03) on microbiota modulation in CD children on a GFD was evaluated by Quagliarello et al. The study population consisted of 40 CD children (randomly allocated into two groups: 20 in the probiotic group and 20 in the placebo group) and 16 healthy children as controls. The authors revealed an imbalance of the intestinal microbial composition of CD patients mainly characterized by a reduction of the *Firmicutes/Bacteroidetes* ratio of *Actinobacteria* and *Euryarchaeota* compared to the controls. The supplementation was able to induce an increase of *Actinobacteria* as well as a restoration of the *Firmicutes/Bacteroidetes* ratio (31).

Klemenak et al. investigate the same mixture of *Bifidobacterium breve* strains (BR03 and B632). The authors randomized 49 CD children (on GFD) in two groups (*Bifidobacterium breve* strains BR03 and B632 vs. placebo) and demonstrate a reduction of TNF-α levels in the probiotic group after receiving *B. breve* for 3 months. However, the effect of the supplementation is not durable: TNF-α levels increased again 3 months after completion of the intervention. The authors did not reveal any difference in IL-10 levels between the two groups (32).

Recently an RCT on 40 children with CD and 16 healthy controls was performed to evaluate the effect of a mixture of two *Bifidobacterium breve* strains (DSM 16604 and DSM 24706). The result showed TNF-α level reduction and reestablishment of the *Firmicutes/Bacteroidetes* ratio after 3 months of probiotic administration (33).

Olivares et al. demonstrate, in patients on GFD, that *Bifidobacterium longum* CECT 7347 led to a reduction in activated T-lymphocytes and TNF-α levels as compared to placebo. Moreover, the increase in height percentile was significantly greater in patients who assumed probiotic compared to those who assumed placebo (*p*<0·048) although weight percentile was similar in the two groups. The probiotic treatment also induced a significant decrease in the *Bacteroides fragilis* and content of sIgA in stools. No differences in GI symptoms were observed between probiotic and placebo groups (34). Our group performed an RCT on 109 CD patients with IBS symptoms despite GFD; we assessed the role of a probiotics mixture made up of five strains of lactic acid bacteria and *Bifidobacteria* [*Lactobacillus casei* LMG 101/37 P-17504 (5x10^9^ CFU/sachet), *Lactobacillus plantarum* CECT 4528 (5x10^9^ CFU/sachet), *Bifidobacterium animalis* subsp. *lactis* Bi1 LMG P-17502 (10x10^9^ CFU/sachet), *Bifidobacterium breve* Bbr8 LMG P-17501 (10x10^9^ CFU/sachet), *Bifidobacterium breve* Bl10 LMG P-17500 (10x10^9^ CFU/sachet)] administered for 6 weeks followed by a follow-up period of 6 more weeks. We demonstrate that the probiotic mixture was able to improve the severity of IBS symptoms. In detail, we showed a higher percentage of treatment success (defined as a decrease of at least 50% of IBS severity score) at both intention-to-treat (14.8% vs. 3.6%; *p*<0.04) and per-protocol analysis (15.3% vs. 3.8%; *p*<0.04) after 6 weeks of treatment. Moreover, the probiotic mixture exerted a positive microbiota modulation with a durable increase of *bifidobacteria* persistent 6 weeks after the end of the treatment (35).

Finally, Harnett et al. studied 45 CD patients complaining of GI symptoms despite strict adherence to GFD for 12 months. Participants were randomized to 5 g of VSL#3 probiotic formulation or placebo for 3 months. The results revealed no statistically significant changes in the fecal microbiota between the groups (36).

Overall, no study has demonstrated that supplementation of probiotics to untreated CD subjects leads to normalization of the gut histology or prevents that changes occur in the mucosal architecture on long term after oral gluten challenge; therefore, all the studies are to be considered preliminary and without an application in the clinical practice.

## Conclusion

Several shreds of evidence demonstrate that probiotics are an excellent resource of endopeptidases for digestion and reduction of gluten toxicity; however, we are far from a possible application in the clinical practice. We firmly believe that, at present, GFD is the only therapeutic option for CD patients and that it must be rigorous and permanent. A future challenge is the possible application of the biochemical machinery of the bacterial endopeptidases to digest the gluten toxic epitopes to be used to produce a wheat gluten-free flour maintaining the nutritional value of this prohibited cereal. The idea that the use of probiotics can allow even minimal transgressions to the diet must be discouraged because, at the moment, there is no data to support this hypothesis.

## Author Contributions 

All authors have participated in drafting and revising the review, and they have seen and approved the final version. All authors take full responsibility for the manuscript. All authors contributed to the article and approved the submitted version.

## Conflict of Interest

RF is the inventor of the patent N 0001425900, released on November 17, 2016 (Italy).

The remaining authors declare that the research was conducted in the absence of any commercial or financial relationships that could be construed as a potential conflict of interest.
